# Impaired proteolysis by SPPL2a causes CD74 fragment accumulation that can be recognized by anti‐CD74 autoantibodies in human ankylosing spondylitis

**DOI:** 10.1002/eji.201948502

**Published:** 2020-04-14

**Authors:** Tessa S. van Kempen, Emmerik F.A. Leijten, Marthe F.S. Lindenbergh, Michel Olde Nordkamp, Christoph Driessen, Robert‐Jan Lebbink, Niklas Baerlecken, Torsten Witte, Timothy R.D.J. Radstake, Marianne Boes

**Affiliations:** ^1^ Department of Rheumatology & Clinical Immunology University Medical Center Utrecht Utrecht The Netherlands; ^2^ Center for Translational Immunology University Medical Center Utrecht Utrecht The Netherlands; ^3^ Department of Biochemistry and Cell Biology Faculty of Veterinary Medicine Utrecht University Utrecht The Netherlands; ^4^ Department of Oncology and Hematology Cantonal Hospital St. Gallen St. Gallen Switzerland; ^5^ Department of Medical Microbiology University Medical Center Utrecht Utrecht The Netherlands; ^6^ Department of Clinical Immunology and Rheumatology Medical University Hannover Hannover Germany; ^7^ Department of Pediatrics University Medical Center Utrecht Utrecht the Netherlands

**Keywords:** Ankylosing spondylitis, Autoimmunity, CD74, Monocytes, SPPL2a

## Abstract

Ankylosing spondylitis (AS) is associated with autoantibody production to class II MHC‐associated invariant chain peptide, CD74/CLIP. In this study, we considered that anti‐CD74/CLIP autoantibodies present in sera from AS might recognize CD74 degradation products that accumulate upon deficiency of the enzyme signal peptide peptidase‐like 2A (SPPL2a). We analyzed monocytes from healthy controls (*n* = 42), psoriatic arthritis (*n* = 25), rheumatoid arthritis (*n* = 16), and AS patients (*n* = 15) for SPPL2a enzyme activity and complemented the experiments using SPPL2a‐sufficient and ‐deficient THP‐1 cells. We found defects in SPPL2a function and CD74 processing in a subset of AS patients, which culminated in CD74 and HLA class II display at the cell surface. These findings were verified in SPPL2a‐deficient THP‐1 cells, which showed expedited expression of MHC class II, total CD74 and CD74 N‐terminal degradation products at the plasma membrane upon receipt of an inflammatory trigger. Furthermore, we observed that IgG anti‐CD74/CLIP autoantibodies recognize CD74 N‐terminal degradation products that accumulate upon SPPL2a defect. In conclusion, reduced activity of SPPL2a protease in monocytes from AS predisposes to endosomal accumulation of CD74 and CD74 N‐terminal fragments, which, upon IFN‐γ‐exposure, is deposited at the plasma membrane and can be recognized by anti‐CD74/CLIP autoantibodies.

## Graphical abstract

Ankylosing spondylitis (AS) patient sera contain anti‐CD74 autoantibodies. Signal peptide peptidase‐like 2a (SPPL2a) is involved in CD74 processing. These results show that a subset of AS patients have impaired SPPL2a enzyme function. SPPL2a dysfunction leads to CD74 fragment accumulation on the plasma membrane, which can be recognized by anti‐CD74 autoantibodies.

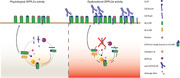

## Introduction

Spondyloarthritis (SpA) and rheumatoid arthritis (RA) are chronic inflammatory rheumatic diseases. The spondyloarthropathies encompass a spectrum of rheumatic diseases subdivided in axial and peripheral SpA that include both ankylosing spondylitis (AS) and psoriatic arthritis (PsA) [1]. SpA is often considered an autoinflammatory condition—caused by repetitive trauma and subsequent inflammatory responses—whereas RA is considered an archetypical autoimmune disease [2, 3]. Although RA and SpA can share features such as destructive and chronic inflammation of the joints, the overall clinical presentation of these diseases is quite distinct. The latter suggests that these conditions share final common pathological pathways, but at the same time have unique molecular mechanisms causative to their specific phenotypes. At the molecular level, both SpA and RA are similar in showing linkage at genome‐wide significance to chromosome 6p21, which harbors the polymorphic human leukocyte antigen (HLA) class I and class II genes [4, 5]. AS is strongly associated with the HLA class I molecule B27, whereas RA is associated with the HLA class II molecule DRB1. The propensity of this genetic background implies that the antigen presentation pathway or the type of T‐cell response elicited is important to the pathogenesis of both diseases.

The initiation of all adaptive immune responses requires the display of antigenic fragments by professional antigen‐presenting cells (APCs) to T cells and B cells. As part of the MHC class II antigen presentation pathway, CD74/MHC class II complexes are sorted from the ER to late endosomal compartments. Cathepsin S is an important protease in this process, cleaving the N‐terminal side of CD74 to produce class II‐associated invariant chain peptide (CLIP), which occludes the HLA class II peptide binding cleft and thereby prevents premature peptide loading. A defect in cathepsin S proteolysis leads to the accumulation of CD74‐p10, retention of the sorting signals in the N‐terminus, interference with peptide loading, and a block in surface‐directed HLA class II transport [6, 7, 8, 9]. Cathepsin S deficiency furthermore shows aberrant endosomal architecture in B cells [7]. Finally, the transmembrane part of CD74 is processed by intramembrane cleavage by the protease signal peptide peptidase‐like 2A (SPPL2a). Disturbed SPPL2a proteolysis triggers the accumulation of CD74‐p8 fragments, enlargement of endosomes, and, in contrast to cathepsin S deficiency, increased plasma membrane display of full‐length CD74 and HLA class II [10, 11, 12]. The rate of CD74 proteolysis, as contributed by cathepsin S and presumably SPPL2a, thereby imparts control over endosomal architecture and surface‐directed transport of CD74 and HLA class II molecules.

IgG autoantibodies against the CD74/CLIP domain are associated with early AS [13, 14, 15, 16]. However, mechanisms contributing to the production of CD74 autoantigens are unknown. We here tested the hypothesis that anti‐CD74 autoantibodies recognize residential products from CD74 that accumulate upon impaired SPPL2a function.

## Results

### Monocytes from AS patients have decreased SPPL2a enzyme activity

**Figure 1:**
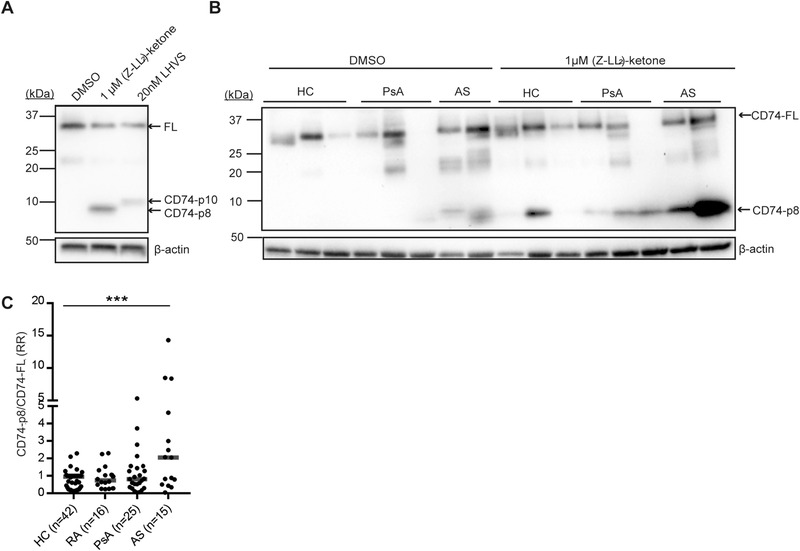
Monocytes from AS patients show an increase in CD74‐p8 accumulation. (A–C) CD14+ monocytes were isolated from PBMCs from HC, RA, PsA, and AS by CD14 magnetic beads. (A, B) CD14+ monocytes were incubated with the vehicle control DMSO, 1 μM (Z‐LL)_2_ ketone, or 20 nM LHVS for 24 h. At least two HCs were included on each Western blot. The protein lysates were tested for CD74 using an antibody directed against the N‐terminus (clone: 2D1B3). The blot was stripped and reprobed for β‐actin as a loading control. Representative blots. (C) CD74‐p8 was normalized to CD74‐p33/p35 (CD74‐FL) following normalization to a HC. Data combined from 16 independent experiments with seven to eight samples per experiment. The median is represented in the dot plot. ***p < 0.001; unpaired *t* test.

We first took a pharmacologic inhibitor‐based approach by culturing monocytes from healthy control (HC) in the presence or absence of the SPP/SPPL inhibitor (Z‐LL)_2_‐ketone for 24 h at the published IC50 dose (1 μM [17, 18, 19]). CD74‐p8 accumulation was tested on Western blot. As a control, we treated monocytes from HC with cathepsin S inhibitor LHVS (20 nM). LHVS blocks the endosomal proteolysis of CD74‐p10 to CLIP [6]. Monocytes were still viable at the concentrations (Z‐LL)_2_‐ketone and LHVS used (data not shown). Confirming earlier results, the SPPL2a inhibitor‐treated monocytes showed N‐terminal fraction (NTF) accumulation of 8 kDa (CD74‐p8) [10, 11, 12], and the cathepsin S inhibitor‐treated monocytes revealed accumulation of CD74‐p10 [6] (Fig. 1A). Thus, both enzymes promote proteolysis of CD74 in human monocytes.

We next examined the SPPL2a enzyme function in monocytes from HC donors and patients diagnosed with PsA, RA, and AS (characteristics of the study population are described in Table 1). Lysates from monocytes were tested for CD74‐p8 accumulation on Western blot. Without pharmacologic inhibition (baseline), some CD74‐p8 accumulation could be detected, but only in patients (Fig. 1B). We first quantified and normalized the full‐length of CD74 (33/35 kDa) and CD74‐p8 to β‐actin in (Z‐LL)_2_‐ketone‐treated monocytes and assessed whether full‐length CD74 correlated with CD74‐p8 accumulation. In RA, full‐length CD74 strongly correlated with CD74‐p8 accumulation (*r* = 0.78, *p* = <0.001) (Supporting Information Fig. 1B). In HC and PsA, we also found a correlate between full‐length CD74 and CD74‐p8 (*r* = 0.44, *p* = 0.004 and *r* = 0.58, *p* = 0.03, respectively) (Supporting Information Fig. 1A and C). In AS, however, we did not observe a correlation between the two measurements (*r* = 0.23, *p* = 0.42) (Supporting Information Fig. 1D). This suggests that the increased accumulation of CD74‐p8 in monocytes from HC, RA, and PsA was caused by increased substrate availability. In AS, CD74‐p8 accumulation is independent of substrate availability. Due to this observation, CD74‐p8 was normalized to full‐length CD74. After full‐length CD74 correction, monocytes from AS patients showed significantly more CD74‐p8 accumulation upon SPPL2a inhibition compared to HC (Fig. 1C). Thus, CD74 proteolysis by SPPL2a is affected in monocytes from AS patients.

**Table 1:** Clinical and demographic characteristics of the study group

Variable	HC (N = 42)	RA (N = 16)	PsA (N = 25)	AS (N = 15)
Age in years – median (range)	45 (23–65)	61 (26–73)	54 (31–73)	42 (19–65)
Female sex – No. (%)	20 (48)	12 (75)	6 (24)	4 (27)
Disease duration in years – median (range)	n.a.	10 (1–28)	11 (1–28)	9 (0.5–30)
Age of disease onset in years – median (range)	n.a.	46 (14–69)	44 (24–66)	33 (13–57)
HLA‐B27+ – No. (%)	n.a.	n.a.	0 (0)	12 (80)
RF+ – No. (%)	n.a.	11 (69)	1 (4)	0 (0)
Axial involvement – No. (%)	n.a.	n.a.	1 (4)	15 (100)
ESR in mm/hour – median (range)	n.a.	7 (5–47)	8 (1–42)	13 (1–42)
TJC, of 76 – median (range)	n.a.	4 (0–13)	1 (0–26)	0 (0–4)
SJC, of 78 – median (range)	n.a.	3 (0–13)	0 (0–13)	0 (0–4)
Systemic immunomodulatory medicationa) – No. (%)	n.a.	16 (100)	18 (72)	3 (20)

RF, rheumatoid factor; ESR, erythrocyte sedimentation rate; TJR, ender joint count; SJC, swollen joint count; BASDAI, Bath Ankylosing Spondylitis Disease Activity Index.

a Systemic immunomodulatory medication consisted of methotrexate (MTX; 10 patients with RA, 16 patients with PsA, one patient with AS), leflunomide (two patients with PsA), hydroxychloroquine (HCQ; one patient with RA), prednisone (one patient with PsA), sulfalazine (SSZ; two patients with AS), MTX + HCQ (five patients with RA), and SSZ + prednisone (one RA patient).

**Figure 2:**
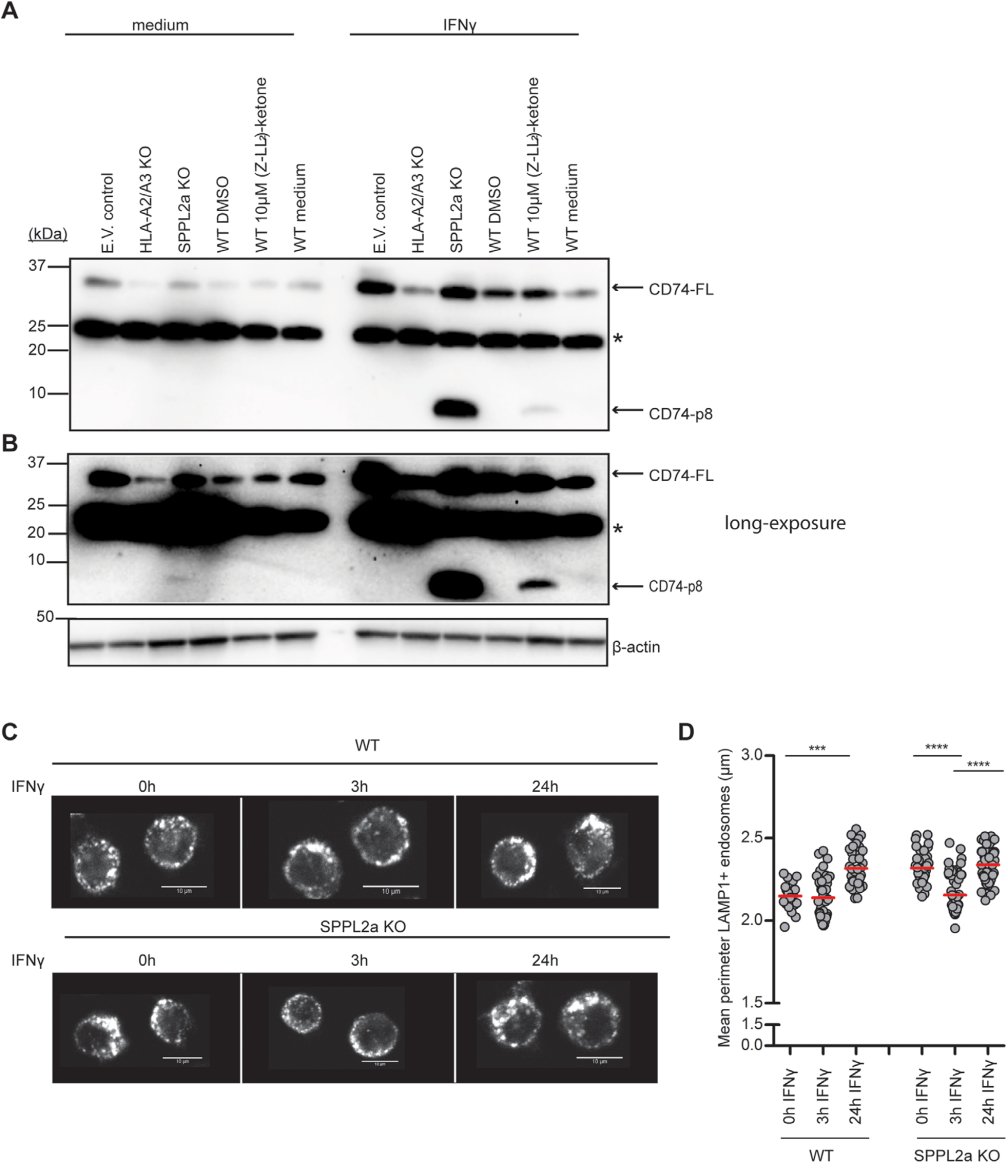
SPPL2a KO cells show compression in endosomal perimeter size upon IFN‐γ stimulation. For the generation of a SPPL2a KO cell line, THP‐1 was depleted for the targets using CRISPR/Cas9 combined with lentiviral transduction technology. Empty vector and HLA‐A2/A3 KO were used as CRISPR controls. (A, B) The effect of CRISPR gene destruction in THP‐1 cells was evaluated on Western blot. The CRISPRed cells were treated with IFN‐γ for 24 h. WT cells were treated with vehicle control DMSO or 10 μM (Z‐LL)_2_‐ketone in the presence or absence of IFN‐γ for 24 h. Western blot lysates were blotted for CD74 N‐terminus (clone: 2D1B3). Blots were stripped and reprobed for β‐actin as a loading control. Blot is representative of three independent experiments. (B) In the long exposure, CD74‐p8 can be detected in the untreated SPPL2a KO THP‐1 lysates. The asterisk (*) represents nonspecific protein binding by the primary antibody. (C) Representative immunofluorescence images of LAMP‐1+ endosomes of WT and SPPL2a KO THP‐1 cells that were treated with or without IFN‐γ for 0, 3, or 24 h. Image captured at 63× magnification. Scale bars: 10 μm. (D) Dot plot represents quantification of the perimeter of LAMP‐1+ endosomes. Eight to nine pictures with at least 20 cells from each condition were taken. Each dot represents the mean endosomal LAMP1+ perimeter from one confocal image, analyzed by the Image J plug‐in Squassh. The data are combined from five independent experiments with one sample condition/experiment. ***p < 0.001; ****p < 0.0001; one‐way ANOVA, Tukey's test.

It appears that there are two groups of AS patients, namely those who have a low accumulation of CD74‐p8 and those who have a high accumulation of CD74‐p8. We examined whether there is a correlation between the accumulation of CD74‐p8 and clinical parameters. No correlation was found between CD74‐p8 and the inflammatory marker erythrocyte sedimentation rate, Bath Ankylosing Spondylitis Disease Activity Index (BASDAI), disease duration, sex, or systemic immunomodulatory medication (Supporting Information Table 1 and Fig. 2A).

### Knockout of SPPL2a results in retention of CD74‐p8 in response to IFN‐γ

To understand the function of SPPL2a in monocytes, we created SPPL2a‐deficient cells using CRISPR‐based whole genome editing in a human monocytic cell line THP‐1. Additionally, we considered that interferon‐gamma (IFN‐γ) plays an important role in the antigen presentation pathway and CD74 processing [20, 21, 22], and therefore tested CD74‐p8 accumulation in SPPL2a wild type (WT), empty vector (E.V.) control, and SPPL2a knock out (KO) THP‐1 cells, in both the absence and presence of IFN‐γ. We found that both untreated and IFN‐γ‐treated SPPL2a KO cells yield CD74 accumulation (Fig. 2A and B). However, IFN‐γ‐treated SPPL2a KO cells showed a higher expression of CD74‐p8. In order to confirm that the NTF of CD74 in IFN‐γ‐stimulated SPPL2a KO cells corresponds to CD74‐p8, we compared the size of the NTF with (Z‐LL)_2_‐ketone‐treated THP‐1 cells. The size of the accumulated NTF in SPPL2a KO THP‐1 cells resembled that of the accumulated NTF in SPPL2a‐inhibited THP‐1 cells, confirming the SPPL2a KO. The SPPL2a KO cells are further confirmed by DNA sequencing (Supporting Information Fig. S3A). These data suggest that the inflammatory signal IFN‐γ increases CD74‐p8 accumulation in THP‐1 cells and we continued our experiments using IFN‐γ‐stimulated SPPL2a KO THP‐1 cells.

### SPPL2A KO THP‐1 cells show compressed LAMP‐1+ endosomes in response to IFN‐γ

To test if the endosomal accumulation of CD74‐p8 drives morphological changes of late endosomes, we measured the average size of endosomes of SPPL2a KO and WT THP‐1 cells. We included IFN‐γ stimulation as a prerequisite condition to examine SPPL2a dysfunction on endosomal morphology in THP‐1 cells. While both WT and E.V. control THP1 cells showed similarly sized endosomes in the steady state, IFN‐γ‐stimulation triggered endosomal enlargement after 24 h (Supporting Information Fig. 4A and B). Pretreatment with cathepsin S inhibitor LHVS resulted in endosomal enlargement [7], which did not enlarge further upon IFN‐γ stimulation. Similarly, SPPL2a KO cells revealed enlarged endosomes at basal level [12] with no additional changes induced after 24‐h IFN‐γ stimulation (Supporting Information Fig. 4A and B). However, SPPL2a KO cells showed compressed endosomes after 3 h IFN‐γ stimulation that return to the original size by 24 h, which is not observed in WT cells (Fig. 2C and D). Overall, these data may suggest that SPPL2a KO cells can resolve the endosomally accumulated CD74 fragments by IFN‐γ‐induced traffic to the surface, after which endosome size reverses to basal level.

### SPPL2A KO THP‐1 cells exhibit increased surface display of HLA‐DR and CD74 in response to IFN‐γ

**Figure 3:**
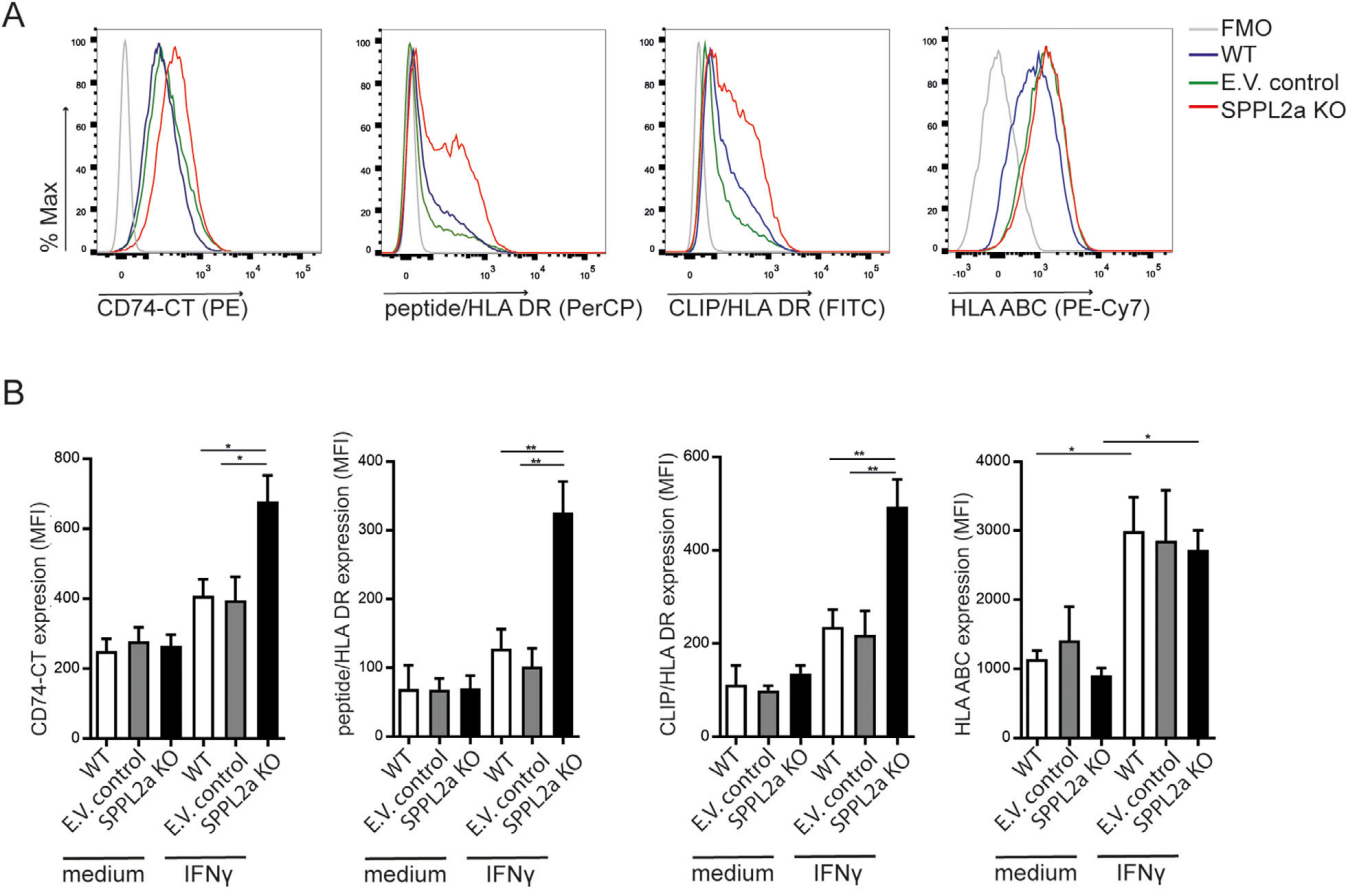
SPPL2a KO cells exhibit increased surface display of full‐length CD74, peptide‐bound HLA‐DR, and CLIP‐bound HLA‐DR upon IFN‐γ stimulation. (A) Representative histogram of flow cytometry analysis of expression of full‐length CD74 C‐terminal (CT; LN2), CLIP‐bound to HLA‐DR (CerCLIP), peptide‐bound HLA‐DR (L243), and HLA‐ABC (G46‐2.6) on WT, E.V. control, or SPPL2a KO THP‐1 cells, incubated in the presence of IFN‐γ for 24 h. (B) Mean fluorescence intensity (MFI) of CD74, CLIP‐bound to HLA‐DR, peptide‐bound to HLA‐DR, and HLA‐ABC on THP‐1 cells from (A). The gating strategies are shown in Supporting Information Fig. 5A. (B) Error bars show mean and SEM from four independent experiments with one sample condition/experiment. The mean is represented in the dot plot. *p < 0.05; **p < 0.01; unpaired *t* test.

To test if SPPL2a deficiency might enhance plasma membrane expression of CD74 and possibly other endosome‐derived molecules, we stimulated THP‐1 cells with IFN‐γ and assessed the cell surface expression of full‐length CD74, peptide‐bound HLA‐DR, CLIP‐bound HLA‐DR, and HLA‐ABC by flow cytometry. Under unstimulated conditions, we observed low levels of CLIP, HLA‐DR, full‐length CD74, and HLA‐ABC in WT, E.V. control, and SPPL2a KO THP‐1 (Fig. 3B). Upon 24‐h IFN‐γ treatment, SPPL2a KO THP‐1 cells showed significantly increased expression of CD74, HLA‐DR, and CLIP‐bound HLA‐DR compared to controls (Fig. 3A and B). We observed no difference in HLA‐ABC surface expression between IFN‐γ‐exposed control and SPPL2a KO cells, confirming that IFN‐γ‐induced HLA‐ABC surface display is not contributed significantly by late endosomal stores [23]. Therefore, the compressed endosomal perimeter in SPPL2a KO associates with increased surface‐directed transport of full‐length CD74, HLA‐DR, and CLIP‐bound HLA‐DR.

### Monocytes from AS patients have increased expression of full‐length CD74 and HLA‐DR

**Figure 4:**
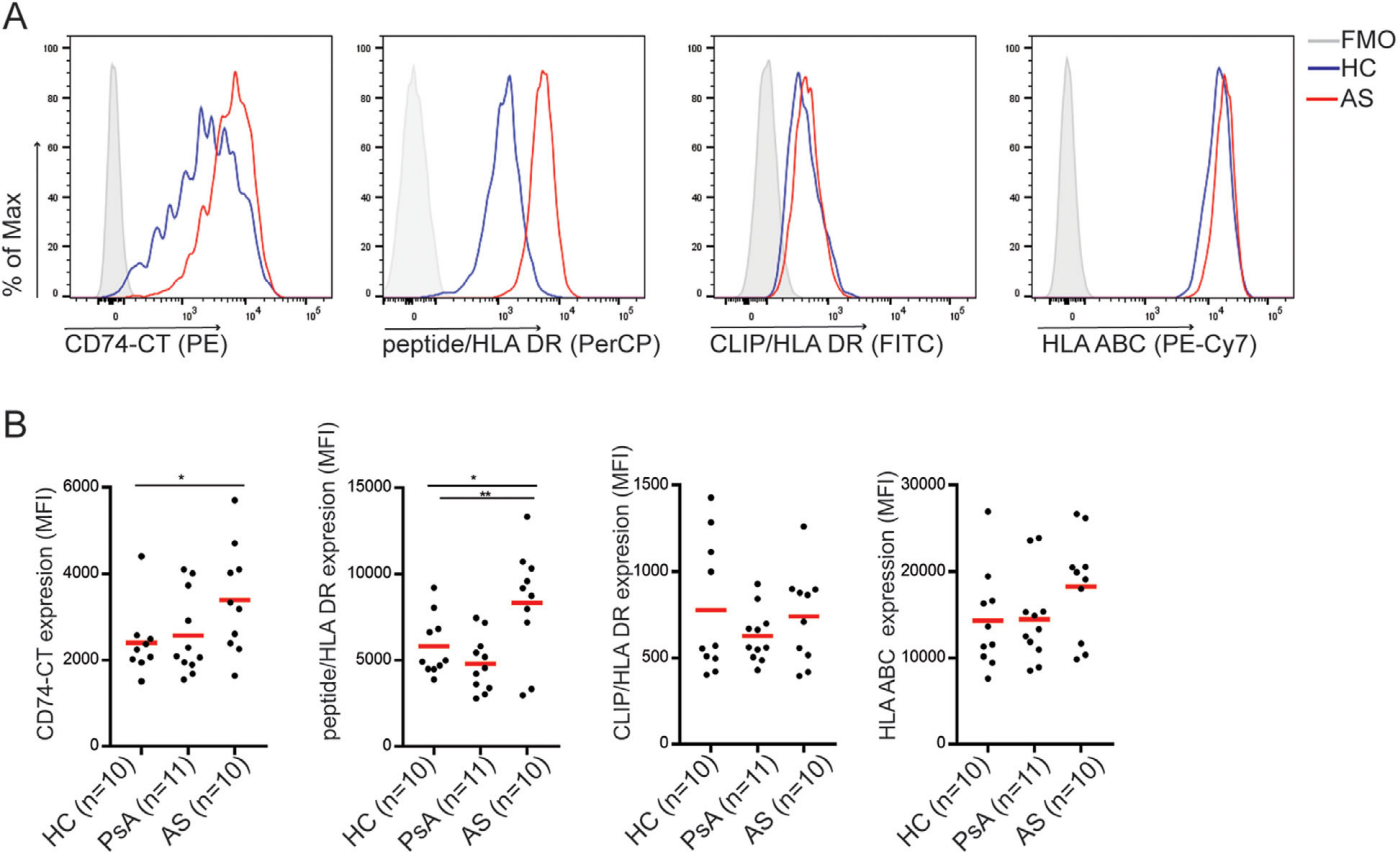
Monocytes from AS patients have increased full‐length CD74 and peptide‐bound HLA‐DR expression. (A) Representative histogram of flow cytometry analysis of expression of full‐length CD74 C‐terminal (CT;LN2), CLIP‐bound to HLA‐DR (CerCLIP), peptide‐bound HLA‐DR (L243), and HLA‐ABC (G46‐2.6) on CD14+ monocytes from HC or AS patient. The gating strategies are shown in Supporting Information Fig. 5B. (B) Dot plot represents the mean fluorescence intensity (MFI) of the cell surface molecules measured from each donor. The mean is represented in the dot plot. Data combined from three independent experiments with three to four HC, PsA, or AS samples per experiment. *p < 0.05; **p < 0.01; unpaired *t* test.

We asked whether defective SPPL2A function in CD14+ monocytes from patients with AS relates to increased cell surface markers. We observed a significant accumulation of full‐length CD74 and peptide‐bound HLA‐DR on the cell surface of AS monocytes, but not in PsA (Fig. 4A and B). CLIP‐bound HLA‐DR and HLA‐ABC were similarly expressed throughout all individuals investigated (Fig. 4A and B). Thus, CD14+ monocytes from AS patients were more prone to increased cell surface expression of HLA class II and full‐length CD74.

### IFN‐γ treatment triggers increased surface display of CD74‐p8 NTF in SPPL2a KO THP1 cells

**Figure 5:**
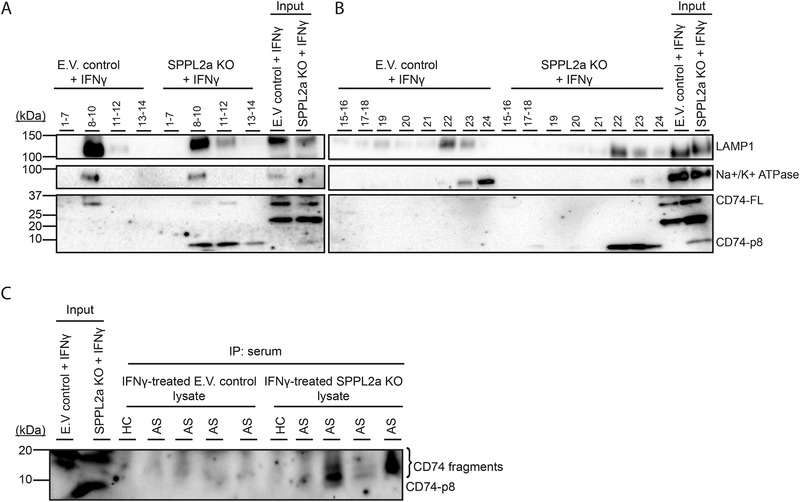
Sera from AS patients contain IgG CD74‐autoantibodies that recognize accumulated CD74 degradation products in SPPL2a KO THP‐1 cells. (A, B) E.V. control and SPPL2a KO THP‐1 cells were stimulated with IFN‐γ for 24 h and lysed in homogenization buffer (HB) and subcellularly fractionated using a Percoll‐density gradient. Twelve fractions were collected with a volume of 500 μL (top fraction 1, bottom fraction 24). Fractions 1–7, 8–10, 11–12, 13–14, 15–16, 17–18, 19–20, 21, 22, 23, and 24 were collected and centrifuged to pellet the organelles and lysed in Laemmli buffer and analyzed for LAMP‐1, CD74 N‐terminus, and Na^+^/K^+^ ATPase expression using Western blot. (C) Protein‐G agarose beads were coated with sera from four AS patients and one HC. IFNy‐treated E.V. control and SPPL2a KO THP‐1 cells were lysed and proteins were immunoprecipitated using serum‐coated beads. Immunoprecipitates were examined using Western blot and analyzed for CD74 fragments. (A–C) Blots are representative of two independent experiments.

To investigate whether NTF (CD74‐p8) can also accumulate on the plasma membrane, we stimulated THP‐1 cells with IFN‐γ for 24 h and isolated subcellular fractions by discontinuous Percoll‐sucrose density gradient, and subjected to Western blot, probing for CD74, LAMP1, and Na^+^/K^+^ ATPase. LAMP1 and Na^+^/K^+^ ATPase were included as markers for late endosome/lysosome and plasma membrane, respectively. As positive controls, whole lysates showed expression of LAMP1, Na^+^/K^+^ ATPase, and both full‐length CD74 and CD74‐p8 (two lanes on the right side of Fig. 5A and B). Both E.V. control and SPPL2a KO cells showed co‐localization of full‐length CD74 with the plasma membrane marker Na^+^/K^+^ ATPase (fractions 8–10, Fig. 5A). E.V. control did not reveal CD74‐p8 accumulation. However, in SPPL2a KO, CD74‐p8 was enriched in both the LAMP‐1, late endosome, fraction (fraction 22, Fig. 5B), and the Na^+^/K^+^ ATPase and LAMP‐1 cell surface fraction (fractions 8–10 Fig. 5A). LAMP‐1 is present in transport vesicles, directed to fuse with the plasma membrane [24]. These data suggest that NTF (CD74‐p8) is transported to the plasma membrane from the late endosomes/lysosomes.

### IgG present in sera from AS recognize CD74 fragments that accumulate in SPPL2a KO THP‐1 cells

We finally investigated whether sera from AS patients contain IgG antibodies directed against CD74 fragments, using IP experiments. We selected sera from four AS patients showing high CD74‐p8 accumulation, and one HC as control. We used lysates from IFN‐γ‐treated E.V. control and IFN‐γ‐treated SPPL2a KO THP‐1 cells as a source of CD74 protein fragments, and screened for the presence of anti‐CD74 antibodies by preincubation of protein G agarose beads with patient or control serum. Immunoprecipitated CD74 fragments, as a measure for the presence of anti‐CD74 antibodies, were examined using Western blot. CD74 fragments were not detected in E.V. control lysates (Fig. 5C). However, we observed anti‐CD74 IgG antibodies in sera from three AS patients, as the antibodies present in patient sera detected CD74 fragments in lysates from SPPL2a KO cells (Fig. 5C). Antibodies that were present in serum samples from HC and one AS patient did not detect CD74 fragments. The molecular weight of the detected CD74 fragments is higher compared to CD74‐p8. This corroborates the finding that auto antibodies recognize CLIP, which is not present in CD74‐p8 [13, 15]. Together, these data support that decreases in SPPL2a function might contribute to the generation of CD74 fragments, which can be recognized by anti‐CD74 autoantibodies.

## Discussion

In this study, we considered that anti‐CD74 autoantibodies present in sera from AS might recognize CD74 degradation products that accumulate upon SPPL2a deficiency. We found defects in SPPL2a function and CD74 processing in a subset of AS patients, which culminated in increased CD74 and HLA class II display at the cell surface. These findings were corroborated by the observations in SPPL2a‐deficient THP‐1 cells, which showed compressed late endosomes upon receipt of an inflammatory trigger. This feature coincided with increased expression of MHC class II and total CD74 at the plasma membrane. Furthermore, the increase in accumulation of CD74 degradation products was recognized by anti‐CD74 autoantibodies present in sera from AS patients. These data reveal a clear distinction between SPPL2a and cathepsin S, as in cathepsin S KO cells the MHC class II molecule expression on the cell surface is impeded and late endosomes are enlarged in size [7, 10‐12]. These results support that cathepsin S proteolysis facilitates MHC class II transport, whereas SPPL2a proteolysis prevents premature MHC class II surface‐directed transport.

It is unclear why there is considerable variation among AS patients, in terms of CD74‐p8 accumulation. No correlation was found when comparing erythrocyte sedimentation rate, disease activity, and duration with CD74‐p8 accumulation. In addition, it is unclear why CD14+ monocytes from PsA did not reveal a significant SPPL2a dysfunction and the presence of anti‐CLIP antibodies, considering that PsA shares some immunologic and phenotypic overlap with AS [13]. Important to note in this respect is that although the heterogeneity in SPPL2a dysfunction is seen in monocytes from AS patients, it supports that causation of the disease and/or the chronic inflammatory state are not singular, and might evolve during the course of the disease.

To our knowledge, this is the first study to describe the increased display of CD74‐p8 at the plasma membrane in response to a defect in SPPL2a activity upon exposure to IFN‐γ. Based on this assertion, the trafficking of MHC class II molecules to the plasma membrane can be accompanied by CD74 remnants. The accumulation of CD74‐NTF at the plasma membrane, which under healthy circumstances would remain hidden from the humoral immune system, might be recognized by autoantibodies directed toward CD74/CLIP and contribute to inflammatory responses in AS [13, 15]. Recently, a potential oral bioavailable inhibitor has been described to significantly and selectively inhibit SPPL2a in mice and rats and as a consequence resulted in a decrease in B cell and mDC numbers [25, 26]. SPPL2a inhibition may suppress autoimmune features, but with the new knowledge that the inhibition of SPPL2a can increase CD74 remnants on the cell surface, the drug can also worsen pathology in patients with AS who are positive for anti‐CD74 autoantibodies.

A limitation of our study is the technical challenge to detect IgG anti‐CD74 antibodies in the serum of AS patients by ELISA, which we attribute to the incompatibility of detergents we used to generate THP‐1 cell lysates as source of CD74 protein fragments, with the antibodies and substrates for ELISA. For this reason, we resorted to IP‐based detection, which allowed for detection of anti‐CD74 reactivity in AS sera and not control sera.

A further limitation is that we did not investigate the cause of the production of anti‐CD74 autoantibodies in AS. In future studies, it would be interesting to examine whether the accumulation of CD74 remnants on the cell surface of SPPL2a‐deficient cells provoke the immune system to produce autoantibodies to CD74, in analogy to viral particles that are kept hidden from the humoral immune system until displayed at the cell surface.

In summary, our data support that a dysfunction in the protease SPPL2a contributes to dysregulation of CD74 processing in AS. Accordingly, exposure to inflammatory stimuli triggers the surface‐directed transport of late endosomal cargo for increased display at the plasma membrane of CD74/CLIP. Our research provides a mechanistic explanation for the production of CD74 autoantigens that can be recognized by CLIP domain‐specific IgG that is seen in 67–85% of AS patients [13, 15].

## Materials and methods

### Ethics

Written informed consent was obtained in all cases before participation, and the study was conducted in accordance with the Helsinki principles. Ethical approval was obtained from the institutional review board in the University Medical Center Utrecht (Study ID NTR4626; 13‐696‐M). Clinical parameters and blood samples were collected from a cohort of patients with AS, RA, and PsA at the outpatient clinic of the Department of Rheumatology and Clinical Immunology. HC blood samples were collected within the UMCU.

### Reagents

(Z‐Leu‐Leu‐NHCH2)2CO, 1,3‐di‐(*N*‐carboxybenzoyl‐ l‐leucyl‐ l‐leucyl)amino acetone, and 2,2′‐(2‐oxo‐1,3‐propanediyl)bis[*N*‐[(phenylmethoxy)carbonyl]‐ l‐leucyl‐ l‐leucinamide) ((Z‐LL_2_)‐ketone) were purchased from Santa Cruz (sc‐311559); *N*‐morpholinurea–leucine–homophenylalanine–vinylsulfone‐phenol (LHVS) was synthesized in the laboratory of Dr. C Driessen, Department of Oncology and Hematology, Cantonal Hospital St Gallen, St Gallen, Switzerland, following protocols as published in [27]. Recombinant IFN‐γ was purchased from eBioscience (34‐8319‐82). The following antibodies were used: mouse anti‐human LAMP‐1 PE (BD; clone H4A3; 555801), mouse anti‐human CLIP FITC (BD; clone CerCLIP; 555981) mouse anti‐human CD74 PE (Biolegend; clone LN2; 326807), mouse anti‐human CD14 Pacific blue (Biolegend; clone M5E2; 301815), mouse anti‐human HLA ABC PE‐Cy7 (BD; clone G46‐2.6; 561349), mouse anti‐human HLA DR PerCP (Biolegend; clone L243; 560896), mouse anti‐human CD74 N‐terminal (Abcam; clone 2D1B3; ab181465), goat anti‐human β actin (Santa Cruz; clone I‐19; SC1616), goat anti‐mouse AF647 (Thermofisher Scientific; A28181), rabbit anti‐human Na‐K ATPase (Cell Signalling; 3010s), mouse anti‐human LAMP1 (BD, clone H4A3; 555798), Rabbit Anti‐mouse‐HRP antibody, (Dako; P0161), and Rabbit anti‐goat HRP antibody (Dako; P0160).

### Monocyte isolation

Peripheral blood mononuclear cells (PBMC) were isolated by Ficoll‐isopaque (GE Healthcare) density gradient centrifugation. CD14+ monocytes were isolated by magnetic cell sorting using mouse anti‐human CD14 magnetic beads and autoMACS Pro Separator (Miltenyi Biotec; 130‐050‐201) according to manufacturer's instructions.

### Cell culture

Human monocytic leukemia cell line (THP‐1) and CD14+ monocytes were cultured in RPMI‐1640 (Gibco) supplemented with GlutaMAX (Fisher Scientific), 100 IU/mL penicillin and 100 μg/mL streptomycin, 20 mM HEPES (Gibco), and 10% fetal calf serum (Biowest). Of note, cultures contained no l‐glutamine, because of its known interference with endosomal acidification and CD74 processing [28]. To overcome the basification of endosomes, the cells were cultured in medium containing Glutamax in all experiments. 293T human embryonic kidney (HEK293T) cells were cultured in Dulbecco's modified Eagle medium (DMEM) supplemented with 10% heat‐inactivated fetal calf serum, glutamax, penicillin, and streptomycin. Mycoplasma infection was tested and ruled on a two‐monthly basis.

### Lentiviral production

HEK293T cells were transfected with the CRISPR/Cas9 vector, pMD.2G, pRSV‐REV, and pMDlg/p‐RRE in order to produce VSVg‐pseudotyped lenti‐CRISPR virions. Viral supernatants were harvested after 48 h, filtered through a 0.45 μm filter and used to transduce THP‐1 cells by infection in the presence of 6 μg/mL polybrene. Cells were centrifuged for 1 h at 700*g*. Transduced cells were selected with 5 μg/mL puromycin (Sigma‐Aldrich) 3 days posttransduction for 48 h. The clones were validated using DNA sequencing. The sequences were analyzed using CRISPR‐ID [29]. The clonally expanded cells were subjected to western blotting to test for their functional defects.

### Transduction of THP‐1 cells using the CRISPR‐Cas9 lentiviral system

A lentiviral CRISPR/Cas9 vector (pSicoR‐CRISPR‐PuroR) is used, which is described previously [30]. The gRNAs were designed based on the reference human genome database. The gene‐specific RNA sequence cloned was: SPPL2a KO: 5′‐GCCGGGGCCGCCCTACTCT‐3′. HLA‐A2/A3: 5′‐GACCTGCGCTCTTGGACCG‐3′. For control KO cells, we used an empty vector.

### Protein extraction and Western blot

For the detection of CD74, 2 × 10^6^ CD14+ monocytes were cultured with the vehicle control DMSO, 1 μM (Z‐LL)_2_‐ ketone, or 20 nM LHVS for 24 h at 37°C. Note that 1 × 10^6^ CRISPRed THP‐1 were either incubated with or without 1000 U/mL IFN‐γ for 24 h at 37°C. CD14+ monocytes and THP‐1 cells were washed twice in PBS, lysed in Laemmli buffer containing 5% β‐mercaptoethanol, and boiled for 5 min. The samples were stored at −20°C until further use. The protein concentration was determined using BCA protein assay kit (Thermo Fisher Scientific; 23225), separated on a 4–20% polyacrylamide gradient gel (Biorad), and transferred on 0.2 μm PVDF‐P^SQ^ (Thermo fisher scientific) membrane by wet immunoblotting. Membranes were blocked with 5% filtered bovine serum albumin (BSA) followed by primary antibody incubation (overnight 4°C in 0.5% BSA/TBS‐T), and stained with secondary antibody (RT, 1 h in 0.5% BSA/TBS‐T). Between each step, the membranes were washed three times in TBS‐T. For the detection of LAMP‐1 and Na‐K ATPase and CD74‐NT in endosomes, endosomes were resuspended in Laemmli buffer without reducing agent and separated on 10% polyacrylamide gel. Protein was transferred on 0.45 μm PVDF‐P membrane and blocked with 5% dried nonfat milk. Primary and secondary antibody steps were performed as described above. The blot was developed using enhanced chemiluminescence (ECL)‐prime (GE healthcare; RPN2236) and detected in the Bio‐Rad Chemi‐doc. To confirm equal protein loading, antibody against β‐actin was used. The bands were quantified using ImageLab, Bio‐Rad.

### Immunofluorescence

Note that 2.5 × 10^5^ THP‐1 cells were seeded in a 24‐well plate and stimulated with 1000 U/mL IFN‐γ for 0, 3, or 24 h at 37°C. Furthermore, THP‐1 cells were incubated with 20 nM LHVS for 24 h at 37°C. After 23.5 h, 20 μM Hoechst was added to each well. Eight‐well Lab‐Tek® II Chamber Slides (ThermoFischer scientific) were precoated with 0.001% Poly‐l‐lysine for 30 min at RT and washed twice with PBS. The Hoechst‐stained THP‐1 cells were plated in the chamber slides and centrifuged for 30 s at 300*g* in order to let the cells adhere to the slides. The cells were fixed with cytofix/cytoperm (BD biosciences) for 30 min at RT. Cytofix/cytoperm was removed and the cells were blocked with 2% goat serum in permeabilization buffer (eBioscience) for 30 min at RT, followed by anti‐LAMP‐1 incubation (45 min, RT) and goat anti‐mouse AF647 secondary antibody (ThermoFisher scientific; 45 min, RT). Between each step, the slides were washed once with permeabilization buffer. Coverslips were mounted with Mowiol solution. All images were obtained with 1.3× optical zoom using “Plan‐Apochromat” 63 × 1.40 oil DIC M27 objective on a Zeiss LSM710, and processed using Zen 2009 software (Zeiss Enhanced Navigation). LAMP‐1‐positive endosome perimeter was analyzed using the ImageJ plug‐in Squassh [31].

### Flow cytometry

Note that 1 × 10^6^ THP‐1 cells and PBMC from HC, PsA, and AS were stained with the viability dye efluor® 780 (eBioscience; 65‐0865‐14) in PBS for 30 min at 4°C, washed in FACS buffer (PBS supplemented with 1% BSA and 0.1% sodium azide), stained for antibodies directed against CD14, CD74, CLIP, HLA‐DR, and HLA‐ABC plus 10% mouse serum for 15 min at 4°C. Cells were washed and acquired on BD Fortessa flow cytometer. Analysis was done with FlowJo. Gating strategies used to examine the expression levels of the markers are illustrated in Supporting Information Fig. 5. The flow cytometry experiments were performed according to the guidelines [32].

### Endosome and plasma membrane isolation by percoll density gradient centrifugation

Endosome and plasma membrane isolation was performed as described previously with minor modifications [33]. Briefly, 50 × 10^6^ THP‐1 cells were incubated with 500 U/mL IFN‐γ for 24 h at 37°C, harvested, and washed twice in PBS. Cells were resuspended in homogenization buffer (250 mM sucrose, 25 mM Tris‐HCl pH 7.4, and 1 mM EDTA and complete protease inhibitor) and disrupted mechanically by five passages through a 25‐gauge syringe needle. A centrifugation step of 1000*g* for 10 min was performed to obtain a postnuclear supernatant. In a 13‐mL tube, 8.7 mL of 20% Percoll solution in homogenization buffer was under layered with 0.5 mL of 65% (w/v) sucrose in Tris‐HCl, pH 7.4. On top, 1 mL of postnuclear supernatant was added. Finally, the tubes were topped off to a volume of 12 mL with PBS. Centrifugation was performed in centrifuge Optima L‐90K and LE‐80K (Beckman Coulter Optima) in SW40 Ti rotor at 40 500*g* for 60 min at 4°C. Fractions of 500 μL were collected from top (fraction 1) to bottom (fraction 24). The fractions were topped off to a volume of 5 mL with PBS and centrifuged in a SW60 Ti rotor (Beckman Coulter) at 100 000*g* for 1 h 5 min. The pellet was resuspended in Laemmli buffer and loaded on Western blot.

### Immunoprecipitation

Note that 5 × 10^6^ THP‐1 cells were cultured with 500 U/mL IFN‐γ for 24 h at 37°C, washed in PBS, and lysed in CHAPS buffer (1% CHAPS, 30 mM Tris‐HCL pH 8.0, 150 mM NaCl) for 45 min on ice and spun down at 13 000*g* for 15 min. THP‐1 lysates were precleared using empty Protein G‐agarose beads (Roche; PROTGA‐RO) for 30 min under constant agitation at 4°C. Protein G‐agarose beads were incubated with human serum for 2 h under constant agitation at 4°C in order to bind to IgG. Next, beads were washes twice in CHAPS buffer and incubated with precleared THP‐1 lysate for 16 h under constant agitation at 4°C. Beads were separated from lysates by centrifugation, washed twice in CHAPS buffer, and resuspended in Laemmli buffer. The samples were boiled and loaded on Western blot.

### Statistics

Cohorts of healthy donors and patients investigated for CD74‐p8 accumulation and cell surface marker expression were analyzed using two‐tailed unpaired *t* tests. Spearman's rank correlation was used to correlate disease activity parameters and full‐length expression of CD74 to CD74‐p8 accumulation. Confocal microscopy results were analyzed using one‐way ANOVA, followed by a Tukey–Kramer test. Biological replicates for each data point are included in the figure legends. Statistical analyses were performed using IBM SPSS statistics 23 (Statistical Package for the Social Sciences). Data were considered statistically significant at *p* value of <0.05.

## Conflict of interest

Authors declare to have no commercial or financial conflict of interest.

**Abbreviations**
AS: ankylosing spondylitis
E.V.: empty vector
HC: healthy control
NTF: N‐terminal fraction
PsA: psoriatic arthritis
RA: rheumatoid arthritis
SpA: spondyloarthritis
SPPL2a: signal peptide peptidase‐like 2A

## Supporting information

Supporting Information.
