# Supplementary material for: Impaired proteolysis by SPPL2a causes CD74 fragment accumulation that can be recognized by anti‐CD74 autoantibodies in human ankylosing spondylitis
Source: Eur J Immunol. 2020 Apr 14;50(8):1209–19. doi: 10.1002/eji.201948502 (PMC7496470; doi:10.1002/eji.201948502)
Supplement: Supplementary file 1 — Supporting Information. [file EJI-50-1209-s001.pdf]

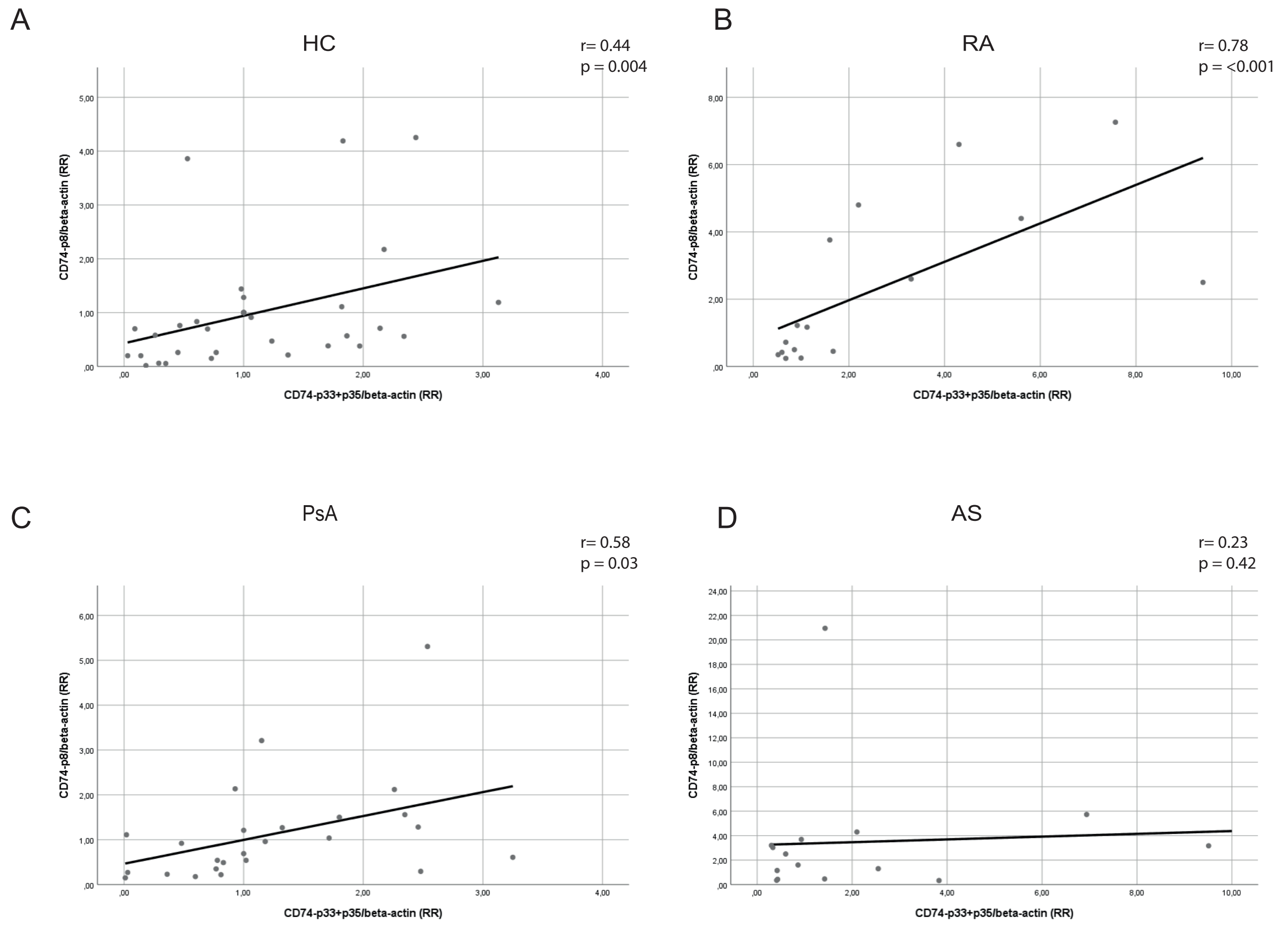

Supporting information Figure S1. CD74-p8 and full-length CD74 (CD74-p33/35) protein expression correlation. (**A**-**D**) CD14+ monocytes were isolated from PBMCs from HC, RA, PsA and AS by CD14 magnetic cell sorter beads. CD14+ monocytes were incubated with 1μM (Z-LL)$_2$ for 24h. At least 2 HCs were included on each western blot. The protein lysates were analyzed for the presence of CD74 protein using an antibody directed against de N-terminus (clone: 2D1B3). The blot was stripped and reprobed for β-actin as a loading control (Sixteen independent experiments). Full-length CD74 and CD74-p8 were normalized to β-actin following normalization to a HC. Full-length CD74 and CD74-p8 were correlated in monocytes from (A) HC, (B) RA, (C) PsA and (D) AS. Spearman's rank correlation (r) and p-value are shown in the figure.

Supplemental information Table 1: Correlation between patient characteristics and laboratory data with CD74-p8.

Clinical variable	*unit*	*N (diagnosis)*	*value*	*p-value*
ESR	mm/hour	14 (AS)	r: 0.24	0.41[a]
Sex	Female/male	15 (AS)	U: 17	0.34[b]
BASDAI	-	11 (AS)	r: 0.43	0.29[a]
Disease duration	Years	15 (AS)	r: 0.12	0.45[a]
Systemic immunomodulatory drugs	-	55 (AS, PsA, RA)	χ^2: 6.282	0.51[c]

[a] Spearman's rank correlation
[b] Mann-Whitney U test
[c] Kruskal Wallis H test
ESR: erythrocyte sedimentation rate; BASDAI: Bath Ankylosing Spondylitis Disease Activity Index.

A

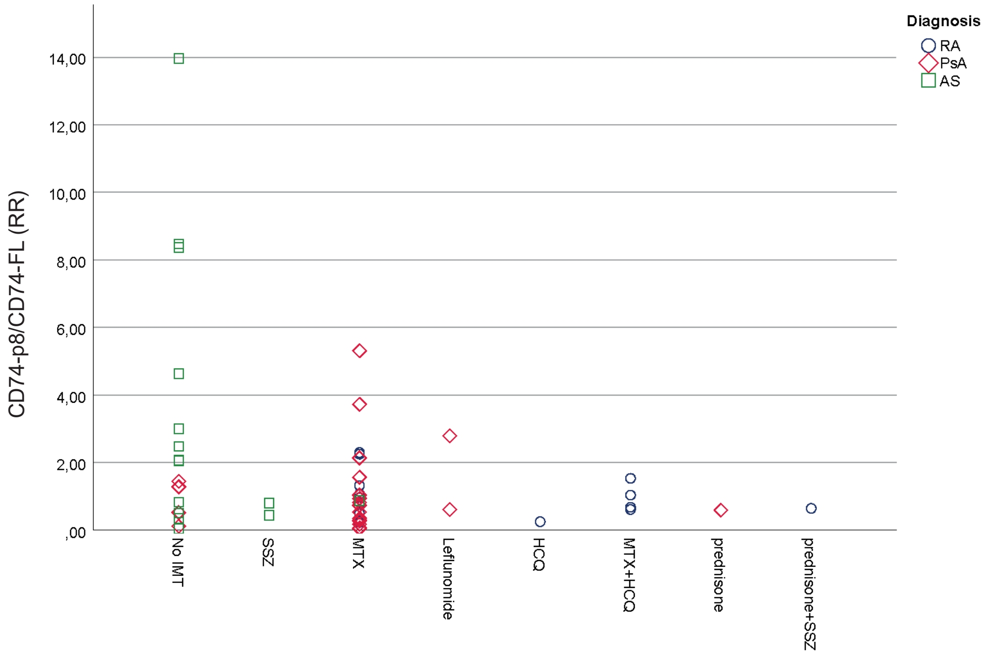

Supporting information Figure S2. (A) CD74-p8 analysis stratified by the usage of systemic immunomodulatory medication. IMT = immunomodulatory therapy, MTX = methotrexate, HCQ = hydroxychloroquine, SSZ = sulfalazine.

A

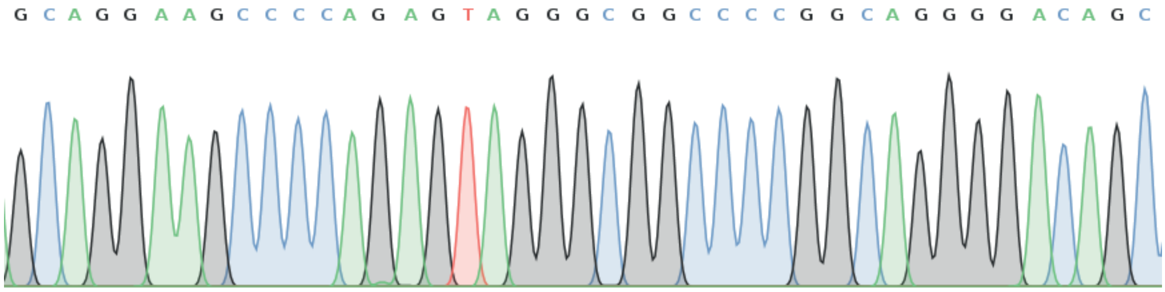

B

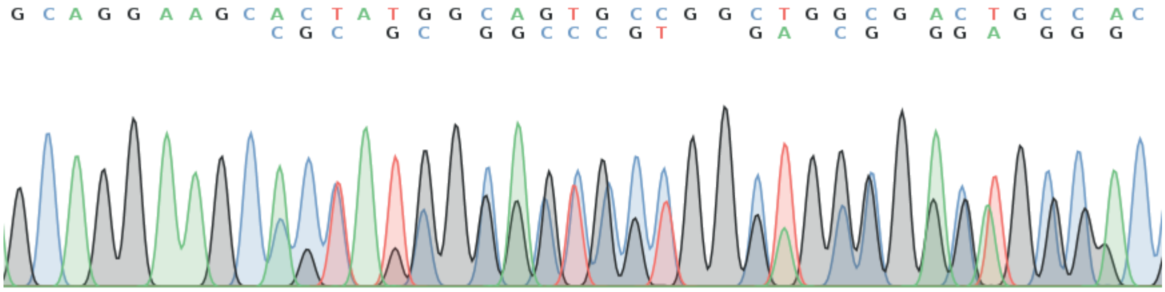

C

```
                          PAM   Guide
WT                  GCCCCAGAGTAGGGCGGCCCCGGCAGGGGACAGCCGCCGCTGCGGCCCCATCGGACT
KO  | allele 1      GCCCCA--------------------------------------------TCGGACT   Δ44
    | allele 2      GC-----AGTAGGGCGGCCCCGGCAGGGGACAGCCACCGCTGCGGCCCCATCGGACT   Δ6
```

Supporting information Figure S3. Sequence analysis of SPPL2a KO cells. Genomic DNA was amplified by PCR and subjected to sequencing. (A) representative sequencing images of WT and (B) SPPL2a KO cells upon CRISPR/Cas9 targeting. The gRNA-target site and PAM sequence is underlined in the figure. (C) Sequence variants of both alleles are depicted.

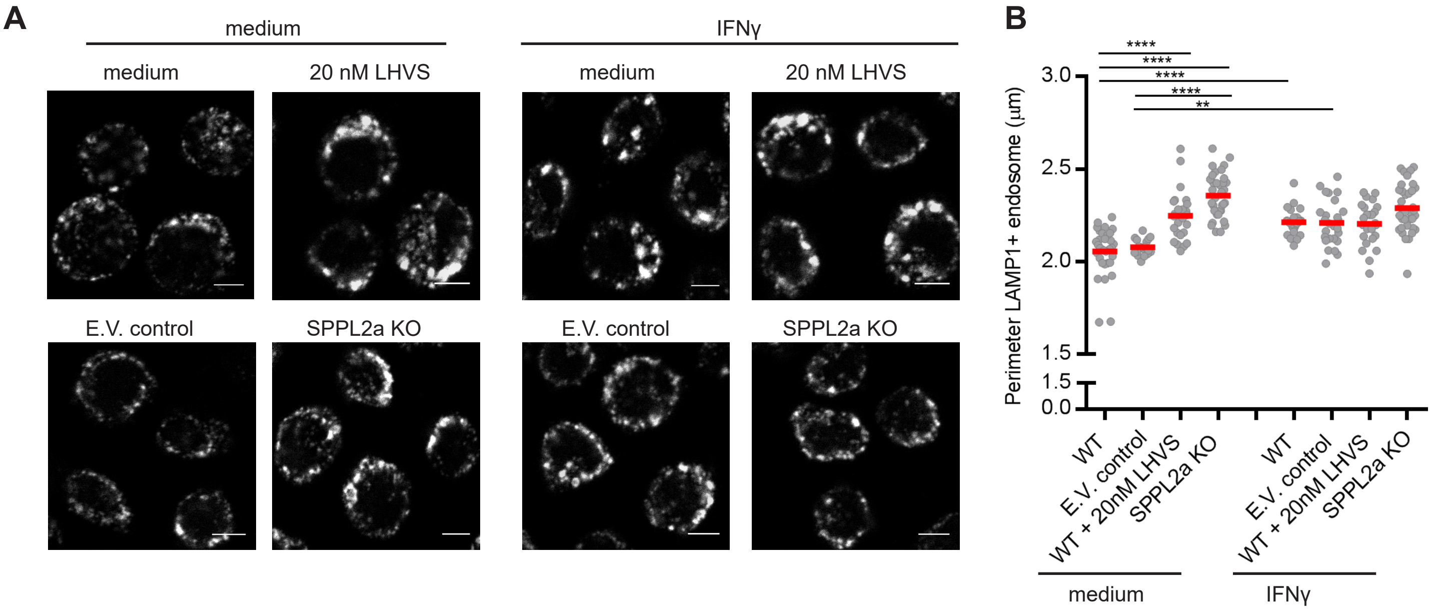

Supporting information Figure S4. SPPL2a KO cells have enlarged late endosomes at baseline. (A) Representative immunofluorescence images of LAMP-1+ endosomes of WT, E.V. control and SPPL2a KO THP-1 cells that were treated with or without IFNγ or with 20 nM LHVS. Image captured at 63x magnification. Scale bars: 10μm. (B) Dot plot represents quantification of the perimeter of LAMP-1+ endosomes. Eight to nine pictures with at least 20 cells from each condition were taken. Each dot represents the mean endosomal LAMP1+ perimeter from one confocal image, analyzed by the Image J plug-in Squassh. The dot plot shows the mean from five independent experiments. *p<0.05; **p<0.01;*** p<0.001; **** p<0.0001; one-way ANOVA, Tukey's test.

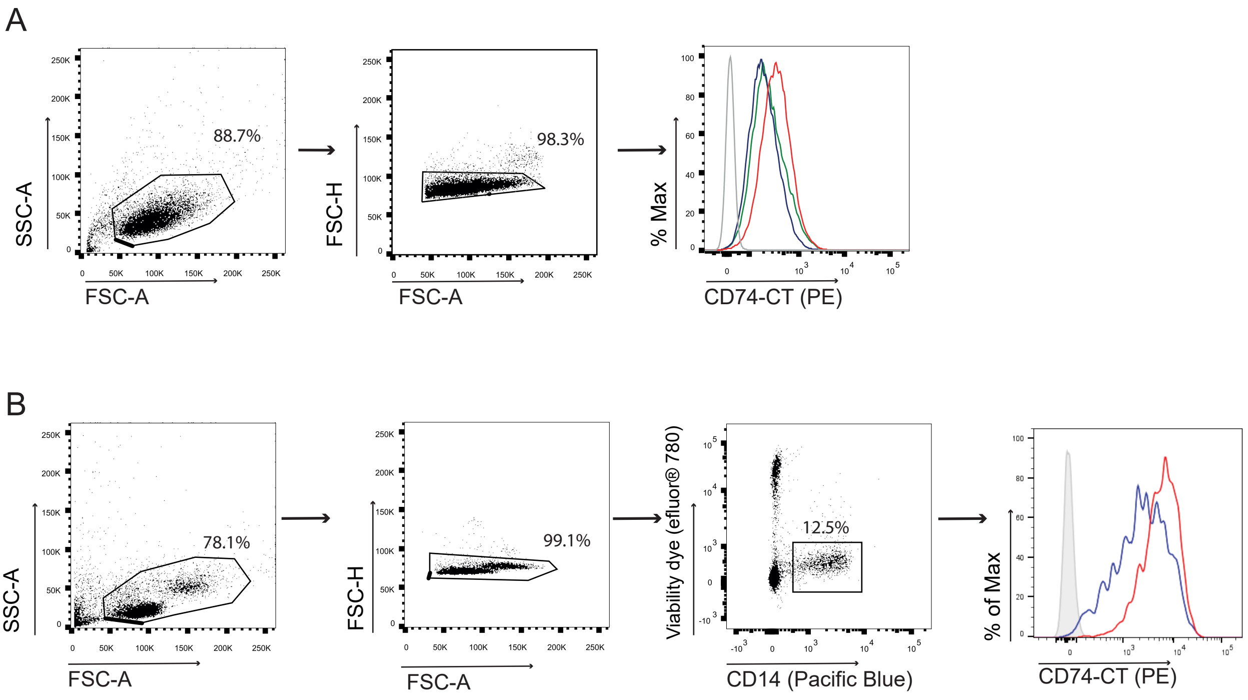

Supporting Information Figure 5. Gating strategy for FACS analysis of THP-1 cells and CD14+ monocytes. (A - B) monocytes and THP-1 cells are processed and cultured according to Material and Methods. (A) THP-1 cells were gated on size and singlets. (B) PBMCs were gated on size, singlets, live and CD14+.
